# Current Challenges of Cardiac Amyloidosis Awareness among Romanian Cardiologists

**DOI:** 10.3390/diagnostics11050834

**Published:** 2021-05-06

**Authors:** Robert Adam, Gabriela Neculae, Claudiu Stan, Ruxandra Jurcut

**Affiliations:** 1 Expert Center for Rare Genetic Cardiovascular Diseases, Department of Cardiology, “Prof. Dr. C.C. Iliescu” Emergency Institute for Cardiovascular Diseases, 022328 Bucharest, Romania; gabriela.neculae94@gmail.com (G.N.); ruxandra.jurcut@umfcd.ro (R.J.); 2 “Carol Davila” University of Medicine and Pharmacy, 030167 Bucharest, Romania; claudiu.stan@drd.umfcd.ro; 3 Department of Nuclear Medicine and Ultrasonography, Fundeni Clinical Institute, 022328 Bucharest, Romania

**Keywords:** amyloidosis, cardiologist, awareness, survey, ATTR

## Abstract

Cardiac amyloidosis (CA) is a restrictive cardiomyopathy characterized by deposition of amyloid in the myocardium and recent studies revealed it is more frequently seen than we thought. Advances in diagnosis and treatment have been made over the last few years that make it desirable to diagnose CA without delay, and that may require extra education. An online survey was conducted among cardiologists from Romania, representing the first assessment of the knowledge of CA among them, with 195 cardiologists answering the questionnaire. There was a wide variation in their knowledge regarding CA. Our participants had limited experience with CA and reported a significant delay between first cardiac symptoms and diagnosis. We address the gaps in knowledge that were identified as educational opportunities in the main identified areas: prevalence and treatment of wild type transthyretin amyloidosis (ATTRwt), prevalence of variant transthyretin amyloidosis (ATTRv) in Romania, diagnosis of CA, the delay in CA diagnosis and available treatment options. Awareness among cardiologists is the most important challenge in diagnosing CA. Romanian cardiologists are partially aware of this topic, but there are still gaps in their knowledge. Educational programs can improve screening of patients with a high suspicion for this progressive condition the prognosis of which has been dramatically changed by the new treatment options.

## 1. Introduction

Cardiac amyloidosis (CA) is a restrictive cardiomyopathy characterized by deposition of amyloid which is represented by misfolded proteins in the myocardial interstitium [1]. The most important classification of systemic amyloidosis is based on the amyloid precursor and although to date there are 36 types of proteins described as precursors [2], CA is determined in most of the cases by two: immunoglobulin light chains and transthyretin.

If the amyloid precursor is represented by misfolded immunoglobulin light chains the disease is called light chain amyloidosis (AL) which is a multisystem disease involving almost any organ and system: soft tissues, cardiovascular system, kidneys, peripheral nervous system, liver or lungs [3]. AL is a rare disease with a reported incidence of approximately 9–14 cases per million person-years [4,5].

Transthyretin amyloidosis (ATTR) results from the accumulation of transthyretin amyloid in the extracellular space of different organs and systems, most commonly in the heart and peripheral nervous system [6]. Normal transthyretin is a tetrameric plasma protein which is responsible for the transportation of thyroid hormones, particularly thyroxine and retinol-binding protein [7]. ATTR manifests as variant ATTR (ATTRv, previously known as hereditary or mutant ATTR) or as wild-type ATTR (ATTRwt, previously known as senile systemic amyloidosis) [8].

ATTRv results from pathogenic mutations in transthyretin gene with an autosomal dominant inheritance pattern and its overall prevalence in Europe is estimated to be less than 1 in 100,000 individuals [7,8]. ATTRv is a heterogenous disorder and its multiorgan involvement and clinical presentation is determined by the pathogenic transthyretin gene mutation: neurological phenotypes, cardiac phenotypes or mixed phenotypes [9].

Distinct from ATTRv, ATTRwt is mainly an isolated CA but it can also affect the soft tissues and the peripheral nervous system leading to bilateral carpal tunnel syndrome or spinal stenosis [10]. ATTRwt is considered a marker of ageing and commonly affects men older than 60 years [11].

As endomyocardial biopsy involves certain risks and may not always be available and the extracardiac biopsy may miss a significant number of patients [12], a non-invasive algorithm [13] represents a valuable alternative to cardiac biopsy for CA by detecting features strongly suggestive of amyloidosis. According to this diagnostic algorithm every patient with a high suspicion of CA should undergo two procedures: a bone-avid, phosphate-based isotope scintigraphy (bone scan) and a search for signs of plasma cell dyscrasia; a positive bone scan combined with the absence of plasma cell dyscrasia sign can diagnose ATTR with specificity and positive predictive value of 100% [13].

Nowadays an increase in the prevalence of CA can be described, particularly in ATTR, as we are witnessing an aged population, an increase in the accuracy of the non-invasive diagnostic algorithm, as well as an increase in the therapeutic options which may require a more accurate diagnosis [8,14]. Given that CA was considered a rare disease and requires a multidisciplinary team, most cardiologists do not have the clinical expertise for diagnosing or treating this disease so misdiagnosis or delayed diagnosis are common. Additionally, despite the revolution in CA treatment with significant advances in the novel therapies, there is a significant number of cardiologists who still believe that this disease has no pathogenic treatment and the only available therapy is represented by the supportive treatment.

In a study involving 1034 patients with CA from the UK National Amyloidosis Centre with a subset of 534 patients in whom complete data on hospital service usage were available, there was a median diagnostic delay from the first presentation with cardiac symptoms of 39 (8–78) months in patients with ATTRwt, with 42% waiting more than 4 years for the diagnosis to be established and a median delay of 25 (4–60) months in the ATTRv group [15]. Another retrospective study involving 270 consecutive patients with CA diagnosed at Toulouse University Hospital reported a median delay between symptoms onset and diagnosis of 8 (5–14), 10 (3–34) and 18 (4–49) months in AL, ATTRwt and ATTRv, respectively, where patients encountered a median number of 2 (1–7) physicians and performed a median number of 3 (1–8) tests before the diagnosis [16].

We performed a survey of the practicing cardiologists from Romania in order to assess their awareness regarding the epidemiology, diagnosis and available treatment options of cardiac amyloidosis, and how this reflects in their practice.

## 2. Materials and Methods

An online survey was conducted among cardiologists from all healthcare centers in Romania in a time interval of 2 months, between 1 September and 31 October 2020. It contained 26 single or multiple choice questions developed by the authors which covered participants’ general data (working place and experience in the field), clinical expertise and particular expertise with CA. The questionnaire was developed based on the authors’ experience, current amyloidosis related consensus documents as well as existing recommendations for survey design [17,18]. A complete copy of the online questionnaire is available in the “Supplementary File”. The survey was introduced on a dedicated platform for online questionnaires and was distributed to a list of cardiologists available to the Romanian Society of Cardiology via email and through social media. The total number of cardiologist members of the Romanian Society of Cardiology is 1475, among which 430 are in training. All participants could respond to the questionnaire only once. Survey participation was anonymous and voluntary and participants were aware that authors were planning to publish the results, thus ethical approval was not required. Data were collected after 2 months and presented as percentages from the total answers.

## 3. Results

### 3.1. Survey Participants

**Figure 1:**
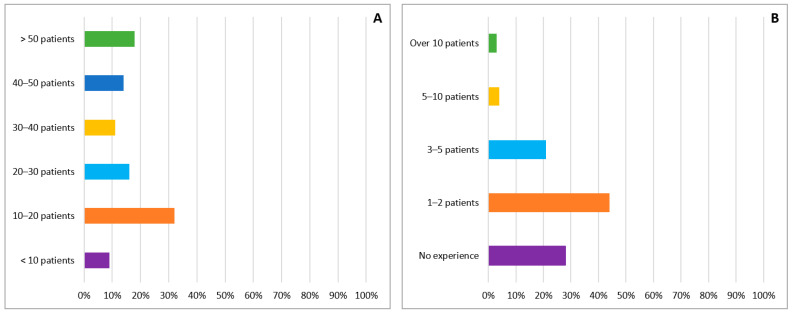
(**A**) Number of general cardiology weekly patients treated by surveyed cardiologists. (**B**) Number of patients presenting with cardiac amyloidosis managed by cardiologists answering the survey.

One-hundred and ninety five Romanian cardiologists answered the online questionnaire. More than half of them (123 participants, 63%) were from three large centers which are distributed symmetrically in the country (Bucharest: 74 participants, 38%; Iasi: 30 participants, 15% and Cluj: 19 participants, 10%). The majority of the participants are working in a University Center (71% vs. 29%) with most of them working on the cardiology ward of a public hospital (139 participants, 72%) followed by private outpatient clinics (29 participants, 15%). The median time for completion of the online questionnaire was 7 min and 41 s with a minimum of 1 min and 58 s and a maximum of 11 h and 39 min. Of the 194 participants who answered this question the majority (178 participants, 92%) described their role as clinical cardiologists, with approximately one third of the participants being subspecialized in: cardiovascular imaging (35 participants, 18%), interventional angiography (12 participants, 6%), heart failure (7 participants, 4%), arrhythmology (4 participants, 2%), rare cardiovascular diseases (3 participants, 1.5%) and other subspecialities (10 participants, 5%). Regarding the training, there was an approximately equal distribution between the three levels of training in Romania: cardiologists in training (65 participants, 33%), specialized cardiologists (60 participants, 31%) and senior cardiologists (70 participants, 36%). The estimated weekly number of patients treated by our surveyed cardiologists is presented in Figure 1A.

### 3.2. Experience with Cardiac Amyloidosis Patients

**Figure 2:**
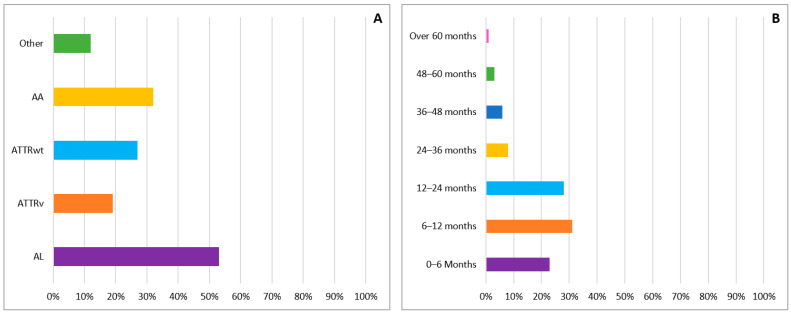
(**A**) Most frequently seen etiologies of cardiac amyloidosis in the clinical practice of the surveyed cardiologists. (**B**) Mean delay from the first presentation with cardiac symptoms to diagnosis of cardiac amyloidosis reported by the surveyed cardiologists.

Almost three quarters of the respondents (141 cardiologists, 72%) stated they had diagnosed or cared for patients with CA in their careers. However, most of the responding cardiologists have little expertise with CA, and very few of them have significant expertise with this disease (Figure 1B). The most common type of amyloidosis seen in our participants practice was AL, followed by ATTR and AA (Figure 2A). The majority of our participants (82%) appreciated the mean delay from the first presentation with cardiac symptoms to diagnosis was between 0 and 24 months and only 10% reported a diagnostic delay of more than 36 months (Figure 2B).

### 3.3. Cardiac Amyloidosis Diagnostic Workup

CA was labeled as a rare disease by approximately three quarters of our surveyed cardiologists (73%). In their view, the main systems and organs affected by systemic amyloidosis were: cardiovascular (99%), renal (85%), peripheral (61%) and autonomic (54%) nervous system, digestive (46%), eyes (14%) and respiratory system (8%). The clinical features of heart failure (HF) that would raise the suspicion of a possible diagnosis of CA in our participants are shown in Table 1.

When specifically questioned on wtATTR, most respondents stated it is a multisystem disease (62%); 35% of cardiologists believe it has a genetic cause and 30% said it can be observed only in people older than 70 years. Almost one half of the respondents know that it can also be identified in a significant proportion of patients with heart failure with preserved ejection fraction (HFpEF) or severe aortic stenosis (48%) and one third of them stated there is no disease modifying treatment available for ATTRwt.

**Figure 3:**
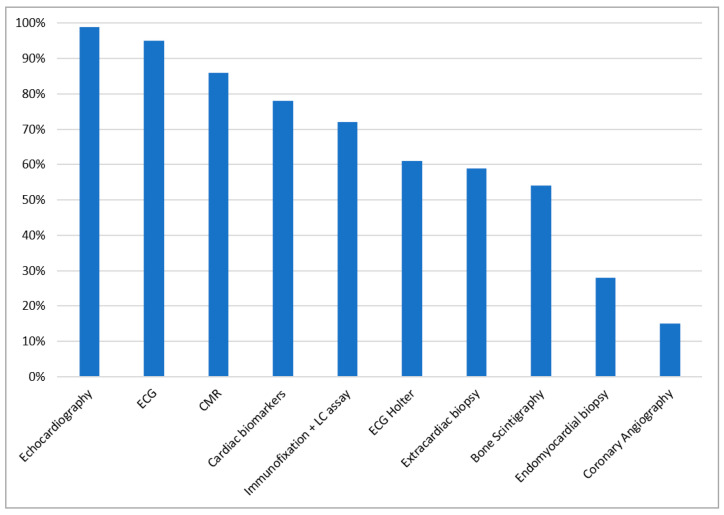
Diagnostic tests considered by the survey participants in patients with heart failure and suspicion of cardiac amyloidosis. CMR cardiac magnetic resonance, ECG electrocardiogram, LC light chain. Cardiac biomarkers are represented by NT-pro BNP and troponin.

**Figure 4:**
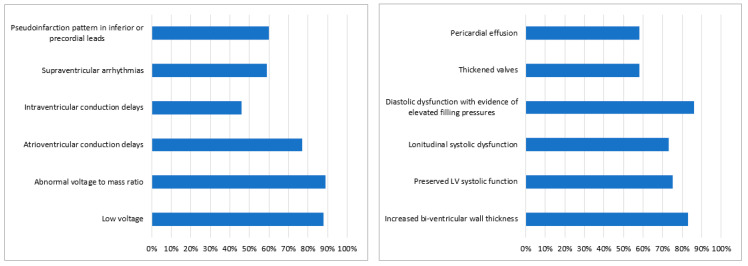
(**A**) Electrocardiographic hallmarks of cardiac amyloidosis chosen by the survey participants. (**B**) Echocardiographic hallmarks of cardiac amyloidosis chosen by the survey participants. LV—left ventricle.

Diagnostic tests considered by our participants in patients with HF and suspicion of CA are shown in Figure 3. When asked about the changes which are mandatorily seen in CA the majority of our participants (45%) answered there are no obligatory changes. Among the other answers, the following hallmarks of CA were chosen as mandatory: restrictive filling pattern (33%), apical sparing pattern on speckle tracking echocardiography (29%), low voltage on electrocardiography (ECG) (28%), concentric left ventricular hypertrophy (LVH) (24%), maximum wall thickness (MWT) of at least 15 mm (21%), postural hypotension (10%), anterior pseudoinfarction on ECG (8%), pericardial effusion (6%) and carpal tunnel syndrome (4%). ECG and echocardiographic features of CA are presented in Figure 4A,B, respectively. When needing to distinguish between the two main types of CA, the preferred investigations were immunofixation and light chain assay (55%), genetic tests (53%) and bone scintigraphy (48%). Other answers were: endomyocardial biopsy (19%), extracardiac biopsy (18%), cardiac magnetic resonance (CMR) (13%), echocardiography (3%) and ECG (2%).

### 3.4. Cardiac Amyloidosis Therapy Awareness

**Figure 5:**
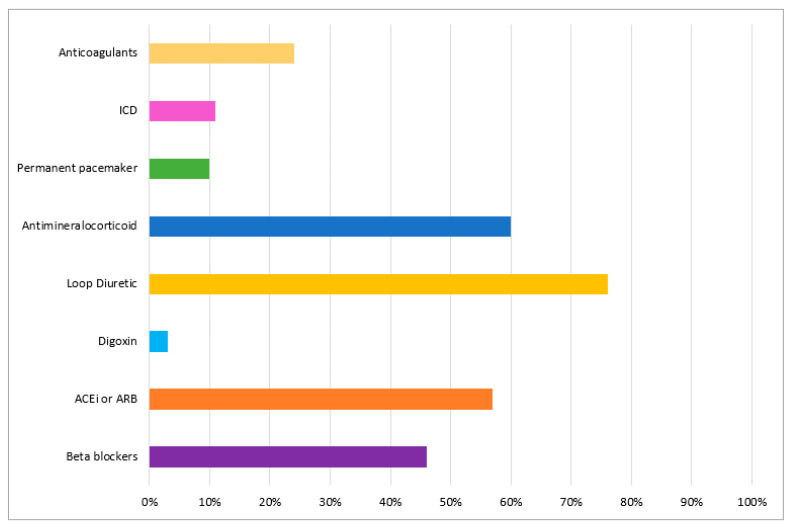
Participants’ options for heart failure treatment in patients with cardiac amyloidosis. ACEi—angiotensin converting enzyme inhibitor; ARB—angiotensin receptor blocker; ICD—implantable cardioverter defibrillator.

In the event of treating patients with suspected CA, 92% of the participants responded they would refer these patients to an expert center, only 22% stated they would initiate the specific treatment and a further 7% said they would continue the HF treatment and would not refer these patients as they did not see the need for further investigations because there is no specific treatment for patients with CA. When asked about HF treatment in this disease most of them had chosen either loop diuretic (75%) or mineralocorticoid receptor antagonists (60%), but a significant proportion stated they would treat these patients with betablockers (46%) and angiotensin converting enzyme inhibitors (57%) (Figure 5). Regarding the disease modifying treatment options most of them had chosen chemotherapy for AL (75%) and tafamidis (73%) or patisiran (35%) for ATTR. Other answers were chemotherapy for ATTR (9%), and tafamidis (8%) or patisiran (9%) for AL (8%).

## 4. Discussion

This survey was conducted at a time when the field of knowledge in cardiomyopathies in general and in CA in particular is in a continuous and rapid development, but the awareness still needs to be improved. As a response to this development of knowledge a scientific statement from the American Heart Association [19] and a position paper from the European Society of Cardiology working group on myocardial and pericardial diseases [20] were published in 2020 and 2021, respectively, by experts in this field. Other important societies, such as the Canadian [21] and German [22] ones recently published their position statements regarding this disease. Unfortunately, there has been no consensus document published by the Romanian Society of Cardiology in this field until now and the diagnosis and management of these patients are based on the European Society of Cardiology and other international societies’ guidelines and recommendations, which the Romanian Society endorses.

This is the first assessment of the knowledge regarding CA among Romanian cardiologists, which highlighted that one of the most important challenges in the diagnosis of CA is awareness. We found a wide variation in cardiologists knowledge regarding CA in general, as well as diagnostic tests and treatment options. The gaps in knowledge should be addressed as educational opportunities in the main identified areas: prevalence and treatment of ATTRwt, prevalence of ATTRv in Romania, diagnosis of CA, the delay in CA diagnosis and available treatment options.

### 4.1. Prevalence and Treatment of ATTRwt

In our survey, both CA in general and ATTRwt were perceived as a rare disease. Moreover, one third of our participants answered ATTRwt can only be observed in people older than 70 years, and only one half of them said that it can be identified in a significant proportion of patients with HFpEF or severe aortic stenosis. However, contrary to common belief, CA is no longer considered a rare disease. Recent studies revealed that ATTRwt can even be considered a common disease, but its prevalence remains to be established. One of the first studies which raised the awareness of this disease was an autopsy study conducted in Finland that showed cardiac transthyretin deposits in nearly one quarter of the 256 subjects older than 85 years [23]. Furthermore, the presence of ATTRwt was not only clinically described in 13% of HFpEF patients above 60 years old and with LVH of more than 12 mm [24], but also histologically described in 14% of the patients with HFpEF enrolled in a more recent study which prospectively evaluated 108 endomyocardial biopsies [25].

Other recent studies demonstrated concomitant ATTRwt in a significant proportion of patients with HFpEF and severe aortic stenosis, with a prevalence of 6–8% in patients with severe aortic stenosis requiring surgical aortic valve replacement [26,27] and a prevalence of 13–16% in patients with severe aortic stenosis referred for transcatheter aortic valve replacement [28,29].

Respondents stated that there is no disease modifying treatment available for ATTRwt, which is currently not correct, as tafamidis was associated with reductions in all-cause mortality and cardiovascular-related hospitalizations and reduced the decline in functional capacity and quality of life as compared with placebo in ATTR-CA patients, and ATTRwt represented three quarters of the ATTRACT study population [30].

### 4.2. Prevalence of ATTRv in Romania

In Romania, a specific mutation with regional distribution for ATTRv (Glu54Gln) was discovered a decade ago [31] and fully characterized more recently [32]. In the latest publication the authors stated 23 patients have been diagnosed with ATTRv in Romania, 18 of whom had the Glu54Gln mutation which is characterized by early disease onset, mixed phenotype (both cardiac and neurological involvement), aggressive disease progression and short survival [32]. Another important finding was that all these 18 patients have origins in the North-Eastern region of Romania (Suceava County) where a cluster of ATTRv patients has been identified [32]. They also reported a prevalence of ATTRv of 1.02 per 1,000,000 people in the general Romanian population with 2.39 per 100,000 people in Suceava County and stated that prevalence of this disease in Romania is substantially underestimated due to several factors, including lack of access to or refusal to receive medical care and limited survival of these patients [32].

Improving knowledge among cardiologists regarding this mixed phenotype form of ATTRv in Romania is of importance, as it can facilitate the timely recognition of this disease in patients who combine LV hypertrophy and sensory–motor neuropathy, especially if originating from this geographical area.

### 4.3. CA Diagnosis

#### 4.3.1. Diagnostic Delay

The majority of our participants stated the mean delay from the first presentation with cardiac symptoms to diagnosis was no longer than 24 months, which is significantly lower than it was previously described in a large study involving patients from the UK National Amyloidosis Centre where a median diagnostic delay of 39 months was reported [15], but consistent with the results from another study involving patients with CA diagnosed at Toulouse University Hospital which reported a median delay between symptom onset and diagnosis of 8, 10 and 18 months in AL, ATTRwt and ATTRv, respectively [16]. Only proper training of healthcare personnel can improve the diagnostic delay in this condition as early diagnosis combined with timely initiation of the disease modifying treatment may lead to disease stabilization and a significant improvement in patient status [19]. For cardiologists, a shortening of the diagnostic delay could be obtained by providing education on the diagnostic tools and the clinical scenarios which characterize CA especially in the early phases, as discussed below. In order to promote proper testing and referrals among general practitioners, who are typically the first point of contact for a newly symptomatic patient, Gertz et al. recently published expert consensus recommendations for the suspicion and diagnosis of transthyretin amyloidosis for the general practitioner [33].

#### 4.3.2. Diagnostic Tools

Timely diagnosis relies on high clinical suspicion, knowledge of cardiac and extracardiac red flags and correct use of diagnostic algorithms. In our survey, around two thirds of cardiologists recognized postural hypotension, neuropathy and carpal tunnel syndrome as red flags for amyloidosis. However, only 53% of respondents mentioned macroglossia and somewhat more than one third would suspect amyloidosis in severe aortic stenosis patients with excessive LVH. Systemic manifestations are frequently seen in ATTR: bilateral sensory–motor polyneuropathy starting in the lower limbs with an ascending pattern and orthostatic hypotension may be present in ATTRv; bilateral carpal tunnel syndrome, lumbar spinal stenosis, and biceps tendon rupture are all common extracardiac manifestations in ATTRwt [34]. Of note, bilateral carpal tunnel syndrome is not only the most common noncardiac manifestation, but can precede clinical HF by several years [35]. A recent study found it present in approximately half of ATTRwt patients 5 to 7 years before diagnosis [36]. While macroglossia is present in 10–15% of AL amyloidosis cases, it has been reported also in few cases of ATTR as well [37].

Interestingly, 59% of cardiologists still rely on extracardiac biopsy and 28% on endomyocardial biopsy in the CA diagnostic workup. However, it is known that the diagnostic yield of fat pad aspirate is only 45% in ATTRv and 15% in ATTRwt [12]. Moreover, a correct and timely CA diagnosis can be based on a non-invasive algorithm [13] which is able to detect suggestive features of CA without the invasive nature and associated risks of endomyocardial biopsy. An essential knowledge for the cardiologist should hence be that every patient with a high suspicion of CA should undergo two procedures: a bone-avid, phosphate-based isotope scintigraphy and a plasma cell dyscrasia workup [13]. Following these two procedures, only if the bone scintigraphy is negative and the search for plasma cell dyscrasia is positive is the extracardiac biopsy needed to confirm the amyloid deposition and to establish the AL diagnosis. In this situation CMR is also very useful to confirm the infiltration of the heart [20]. In the absence of a detectable monoclonal protein the sensitivity and specificity of a bone scintigraphy (grade 2 or grade 3) for ATTR has been proposed to be almost 100% [13] so there is no need for a histological confirmation of amyloid deposits in this case. If the cardiac uptake is grade 1, non-invasive diagnosis is not possible and the histological confirmation and identification of amyloid deposits is required, followed by CMR confirmation of cardiac infiltration if an extracardiac biopsy was used [20].

Survey participants were aware of the clinical scenarios and red flags which should raise the suspicion of this condition as well as the diagnostic tests which are needed to establish the final diagnosis, but only half of them know how to distinguish between the two main types of CA using the non-invasive diagnostic algorithm based on the bone scintigraphy and the search for plasma cell dyscrasia [13]. According to a trend toward multimodality imaging education in the last few years, 86% of cardiologists would chose CMR in the diagnostic workup, as compared to only 54% bone scintigraphy.

Surveyed cardiologists appeared to be aware of the ECG and echocardiographic hallmarks of CA in accordance with the current data from the literature [38]. Furthermore, many cardiologists consider that several features are mandatory in CA on which they can rely for the diagnosis. This is a misconception, as Gonzalez-Lopez et al. showed in a study published in 2017 involving 108 patients with ATTRwt that the clinical spectrum of this disease is heterogeneous and it appears that there is no reliable classic phenotype [10].

#### 4.3.3. Therapeutic Options

The treatment of systemic amyloidosis has dramatically evolved over the last few years in both AL and ATTR, improving disease prognosis. However, treating CA associated HF is a complex task due to the frequent association between systemic congestion and low blood pressure, thromboembolic and hemorrhagic risk balance, patient frailty and poor prognosis. Diuretics represent the first line of medical treatment in HF and CA, which is also reflected in the respondents’ views. However, the cardiologists’ answers in our survey also included betablockers (46%) and vasodilators (57%) as possible therapeutic options, probably extrapolating from conventional HF therapy. However, betablockers are not well tolerated in restrictive cardiomyopathies in general, and CA in particular, while due to sometimes severe postural hypotension, vasodilators have also a very restricted use [19,20]. Anticoagulation was deemed necessary by 24% of the respondents; this is in line with the concepts that CA is often associated with biatrial enlargement and atrial fibrillation, but also that patients with CA present a high frequency of intracardiac thrombosis, especially those with the AL type, even in the presence of sinus rhythm and preserved systolic function, likely because of a poor atrial function [39].

The main advancements in CA therapy come from the development of TTR stabilizers (e.g., Tafamidis) and TTR protein silencers (e.g., patisiran, inotersen). In the ATTR-ACT randomized trial including patients with ATTRwt or ATTRv CA, tafamidis was associated with a significantly lower all-cause mortality (29.5% versus 42.9%) and lower cardiovascular-related hospitalization (0.48 versus 0.70 per year) after 30 months [30] compared to placebo. Subsequently, a study which aimed to assess the clinical characteristics of ATTR in a real-life population was published by the group of the French national center for CA and showed an association between tafamidis treatment and a lower occurrence of cardiovascular outcomes [40]. Other therapeutical options are represented by genetic silencers such as patisiran, which improved multiple clinical manifestations of ATTRv [41] or inotersen which improved the course of neurologic disease and quality of life in patients with ATTRv [42] in phase 3 clinical studies. The latest European recommendations stated that tafamidis should be generally considered the agent of choice in ATTR cardiac patients with reasonable expected survival, while patisiran could be considered in ATTRv patients with cardiac involvement in whom gene silencers are prescribed due to symptomatic neurological disease [20]. Almost three quarters of Romanian cardiologists have knowledge of Tafamidis as a specific therapy for ATTR associated CA (73%), while less than one third (35%) consider patisiran an option in this setting, which is in accordance with the more scarce data supporting this. There is, however, confusion for some cardiologists between the available therapeutic options, with 8% of the respondents believing that these drugs also treat AL amyloidosis.

Conversely, specific treatment in cardiac AL amyloidosis is recommended to be undertaken by multidisciplinary teams involving oncohaematology and cardiology specialists and, whenever possible, patients should be referred to specialized centers [20]. The discovery of bortezomib represented a treatment revolution in achieving rapid hematological responses in patients with AL [43]. The most commonly used first-line treatment is represented by a combination of three drugs—cyclophosphamide, the reversible proteasome inhibitor bortezomib and steroids such as dexamethasone (CyBorD), with weekly administration.

We strongly believe that appropriate education and patients’ screening should now be mandatory to optimize patient care, especially with the significant progress made in this disease’s diagnosis and treatment.

## 5. Study Limitations

This is a small size survey designed to provide information regarding current knowledge on CA among cardiologists from Romania. An important limitation of this study could be that only a small proportion of Romanian cardiologists answered the survey, therefore the results may not be representative of all heart specialists from Romania. However, our respondents were from both academic and non-academic health institutions, covering equally all three stages of training and all of our country’s geographic areas, making this an illustrative sample of medical professionals with relevant answers.

## 6. Conclusions

Awareness among cardiologists is the most important challenge in diagnosing CA. Romanian cardiologists are partially aware of this topic, but several misconceptions and gaps in knowledge exist. Therefore, more educational efforts are needed regarding the latest concepts. This can improve the clinical suspicion, with timely access to screening algorithms for this rapidly progressive condition whose evolution has been dramatically changed by the new treatment options.

## Figures and Tables

**Table 1:** Clinical features of heart failure that would raise the suspicion of a possible diagnosis of cardiac amyloidosis in our participants; EF—ejection fraction, HF—heart failure, LVH—left ventricular hypertrophy.

Answers	Number of Participants	Percentage of Participants
Preserved or reduced EF according to the ESC guidelines for heart failure	140	73%
History of bilateral carpal tunnel syndrome	133	69%
Sensory or motor neuropathy	133	69%
Postural hypotension	130	68%
Nephrotic syndrome	115	60%
Macroglossia	102	53%
Digestive motility disorders	93	48%
HF symptoms with poor response or worsening to standard HF treatment	84	44%
Spontaneous cutaneous ecchymosis	81	42%
Advanced age	72	38%
Severe aortic stenosis and severe, unexplained LVH on imaging	70	37%
Difficulty urinating	30	16%
Lumbar spinal stenosis	23	12%

## Data Availability

The data presented in this study are available on request from the corresponding author.

## Supplementary Materials

The copy of the complete online questionnaire is available online at https://www.mdpi.com/article/10.3390/diagnostics11050834/s1.
