# Supplementary material for: Current Challenges of Cardiac Amyloidosis Awareness among Romanian Cardiologists
Source: Diagnostics (Basel). 2021 May 6;11(5):834. doi: 10.3390/diagnostics11050834 (PMC8148147; doi:10.3390/diagnostics11050834)
Supplement: Supplementary file 1 [file diagnostics-11-00834-s001.zip › diagnostics-1176557-supplementary.pdf]

Supplementary Materials:
Online questionnaire. 1-12 and 26 were single choince questions and 13-25 multiple choice questions.

1. Are you a cardiologist?
    a. Yes
    b. No

2. What is your gender?
    a. Male
    b. Female

3. Choose the region of the country you are working in.
    A list with all the 41 counties of Romania and the Capital City Bucharest was shown

4. Your main working place is:
    a. Public outpatient clinic
    b. Public cardiology department
    c. Private outpatient clinic
    d. Private cardiology department

5. Is your working place a University Center?
    a. Yes
    b. No

6. You are specialized in:
    a. Clinical cardiology
    b. Cardiovascular imaging
    c. Heart failure
    d. Angiography
    e. Electrophysiology and arrhythmia
    f. Rare cardiovascular diseases
    e. Other

7. What is your level of training?
    a. Cardiologist in training
    b. Specialized cardiologist
    c. Senior cardiologist

8. How many patients do you see in average per week?
    a. Bellow 10
    b. 10-20
    c. 20-30
    d. 30-40
    e. 40-50
    f. Over 50

9. Have you ever seen a patient with cardiac amyloidosis?

a. Yes
b. No

10. If yes, how many?
a. 1-2
b. 3-5
c. 5-10
d. Over 10

11. Do you consider cardiac amyloidosis is a rare disease?
a. Yes
b. No

12. Choose an average delay from the first cardiac symptoms to diagnosis in your cardiac amyloidosis patients:
a. 0-6 months
b. 6-12 months
c. 12-24 months
d. 24-36 months
e. 36-48 months
f. 48-60 months
e. 60-72 months

13. What was the final diagnosis in your patients with cardiac amyloidosis?
a. Light chain amyloidosis (AL)
b. Variant transthyretin amyloidosis (ATTRv)
c. Wild type transthyretin amyloidosis (ATTRwt)
d. Inflammatory amyloidosis (AA)
e. Other

14. Please note the organs and systems which can be affected by systemic amyloidosis:
a. Cardiovascular system
b. Peripheral nervous system
c. Autonomic nervous system
d. Digestive system
e. Kidneys
f. Lungs
g. Eyes

15. Which are the characteristics that raise suspicion of cardiac amyloidosis?
a. Heart failure with preserved/reduced ejection fraction according to the ESC guidelines for heart failure
b. Heart failure symptoms aggravated by the conventional treatment
c. Neuropathy with sensory or motor impairment
d. History of bilateral carpal tunnel syndrome
e. Spinal stenosis
f. Macroglosia

g. Hypotension, especially orthostatic hypotension
h. Digestive dysmotility
i. Difficulty urinating
j. Nephrotic syndrome
k. Spontaneous cutaneous ecchymosis
l. Advanced age
m. Association with severe aortic stenosis and severe left ventricle hypertrophy on imaging

16. What diagnostic tests would you recomment for patients with suspected amyloidosis?
a. ECG
b. Echocardiography
c. Cardiac magnetic resonance
d. Bone scintigraphy
e. Cardiac serum biomarkers: NtproBNP, Troponin
f. 24 hours ECG Holter
g. Coronary angiography
h. Extracardiac biopsy
i. Endomyocardial biopsy
j. Genetic tests
k. Serum protein electrophoresis and immunofixation
l. Serum and urinary light chain assessment

17. Which are the ECG hallmarks of cardiac amyloidosis?
a. Low voltage
b. Abnormal voltage to mass ratio
c. Atrioventricular conduction delays
d. Intraventricular conduction delays
e. Supraventricular arrhythmias association (e.g. atrial fibrillation or flutter)
f. Q waves in inferior leads or poor R wave progression in the precordial leads

18. Which are the echocardiographic hallmarks of cardiac amyloidosis?
a. Biventricular hypertrophy
b. Normal global LV systolic function
c. Abnormal longitudinal systolic function
d. Diastolic dysfunction with signs of elevated filling pressures
e. Thickened valves
f. Pericardial effusion

19. What changes are mandatory in patients with cardiac amyloidosis?
a. Bilateral carpal tunnel syndrome
b. Orthostatic hypotension
c. ECG with low voltage
d. Pseudoinfarction pattern on ECG
e. Maximum wall thicknes > 15 mm
f. Concentric hypertrophy
g. Restrictive filling pattern
h. Pericardial effusion

i. Apical sparing pattern of the bull's eye view
J. None of the above

20. What diagnostic tests are mandatory to establish the diagnosis of cardiac amyloidosis?
a. ECG
b. Echocardiography
c. Cardiac magnetic resonance
d. Bone scintigraphy
e. Cardiac serum biomarkers: NTproBNP, Troponin
f. Coronary Angiography
g. Extracardiac biopsy
h. Endomyocardial biopsy
i. Genetic tests
j. Serum protein electrophoresis and immunofixation
k. Serum and urinary light chain assessment

21. What are the tests which can distinguish AL from ATTR cardiac amyloidosis?
a. ECG
b. Echocardiography
c. Bone scintigraphy
d. Extracardiac biopsy
e. Endomiocardial biopsy
f. Genetic tests
g. Serum protein electrophoresis and immunofixation
h. Serum and urinary light chain assessment

22. Choose the characteristics of wild type transthyretin cardiac amyloidosis:
a. It is an isolated cardiac disease
b. Could present as a multi-organ disease
c. It is caused by a transthyretin gene mutation
d. It can be observed only in people older than 70 years
e. It does note have a specific treatment
f. It can be identified in over 10% of patients with HFpEF or severe aortic stenosis

23. What do you recommend to this patients?
a. Specific treatment initiation
b. Referral to a center of expertise
c. Systemic evaluation
d. To continue the heart failure treatment because there is currently no specific treatment available

24. What kind of treatment would you routinely recommend to these patients?
a. Beta blockers
b. Angiotensin-converting enzyme inhibitors or sartans
c. Digoxin
d. Loop diuretics
e. Mineralocorticoid receptor antagonists

f. Permanent pacemaker
g. Implantable cardioverter defibrillator
h. Anticoagulants

25. Choose the right disease modifying treatment for cardiac amyloidosis:
    a. Chemotherapy for AL
    b. Chemotherapy for ATTR
    c. Tafamidis for AL
    d. Tafamidis for ATTR
    e. Patisiran for AL
    f. Patisiran for ATTR

26. Would you be interested in informative materials and scientific events regarding this field?
    a. Yes
    b. No
